# Efficacy of moxidectin, using various dose regimens, against JYD-34, a macrocyclic lactone resistant isolate of *Dirofilaria immitis*

**DOI:** 10.1186/s13071-024-06149-0

**Published:** 2024-04-04

**Authors:** Elizabeth M. Martin, Elizabeth B. Mitchell, Stephen Yoon, John W. McCall, Becky Fankhauser, Abdelmoneim Mansour, Scott McCall, Matthias Pollmeier

**Affiliations:** 1 Boehringer Ingelheim Animal Health, 1730 Olympic Drive, Athens, GA 30601 USA; 2 TRS Labs, Inc, 215 Paradise Blvd, Athens, GA 30607 USA; 3 Boehringer Ingelheim Vetmedica GmbH, Binger Str. 173, 55216 Ingelheim Am Rhein, Germany

**Keywords:** Canine, Macrocyclic lactone, Moxidectin, Heartworm, *Dirofilaria immitis*, JYD-34, Prevention, Resistance

## Abstract

**Background:**

Macrocyclic lactones (MLs) are the only class of drugs currently commercially available that are effective for preventing heartworm disease. The data presented in this article provide information on the efficacy of oral moxidectin against JYD-34, a known ML-resistant *Dirofilaria immitis* isolate, when dogs are treated under various dosing regimens.

**Methods:**

Fifty-two purpose-bred Beagle dogs were used in five laboratory studies. All dogs were inoculated with 50 *D. immitis* third-stage larvae (L_3_) (JYD-34 isolate) 30 days prior to the first treatment. Dogs were randomized to treatment (four to five animals in each group) with one, three, or five monthly doses of oral moxidectin ranging from 6 to 100 µg/kg body weight. In each study, control dogs were not treated. Five to 6 months after L_3_ inoculation, dogs were euthanized, and adult worms were counted to evaluate efficacy of the dosing regimens.

**Results:**

Adult heartworms were recovered from all control dogs, with an overall geometric mean of 29.7 worms (range 15.2 to 38.0, individual counts ranged from 8 to 51). Five monthly doses of 6 µg/kg provided 83.3% and 90.2%, efficacy, and the same number of monthly doses of 9 µg/kg demonstrated 98.8% and 94.1% efficacy. Three monthly doses of 30 and 50 µg/kg demonstrated 97.9% and 99.0% efficacy, respectively, while a single dose of 100 µg/kg demonstrated 91.1% efficacy.

**Conclusions:**

Five monthly doses of 9 µg/kg provided similar or only marginally lower efficacy against JYD-34, a known ML-resistant isolate, compared to substantially higher doses administered for 3 months. This underscores the importance of duration of exposure to moxidectin when facing ML-resistant isolates. Repeated administration of lower doses of moxidectin are an alternative to higher doses in the prevention of heartworm disease associated with less susceptible or resistant isolates.

**Graphical Abstract:**

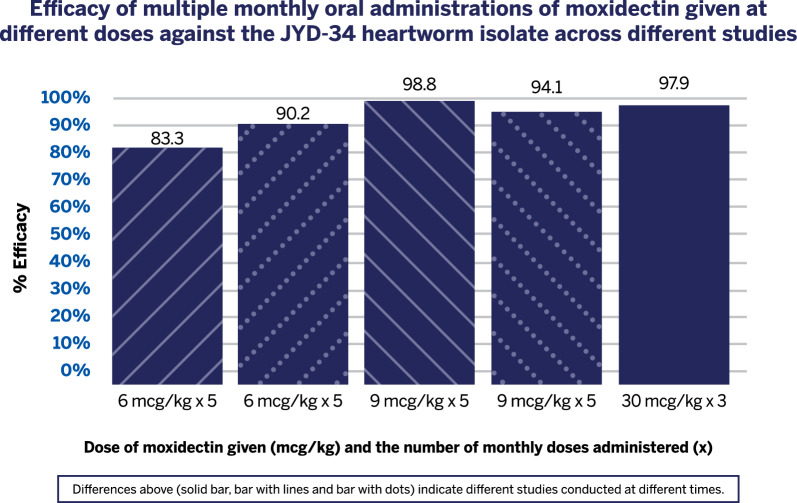

## Background

Heartworm disease, caused by the filarial nematode *Dirofilaria immitis*, is a chronic and potentially life-threatening cardiopulmonary disease of canids with worldwide distribution [1, 2]. Mosquitoes serve as the intermediate host for *D. immitis* and transmit infections not only to the definitive host, wild and domestic canids, but to other species as well, including felids and mustelids [3, 4]. Due to the severity of disease and extensive distribution [4–8], prevention of heartworm disease remains a key focus in companion animal health.

The focus on prevention, rather than treatment, of heartworm disease is related not only to the extensive cardiopulmonary damage that may result from the physical presence of both live and dead nematodes [1, 9], but also to the challenges (e.g., prolonged drug treatment, exercise restriction, lung pathology and potential complications) of adulticidal treatment with melarsomine dihydrochloride, the only drug currently approved for this purpose [2, 9].

At this time, macrocyclic lactones (MLs) remain the only class of commercially available drugs proven safe and effective in preventing heartworm disease. One mechanism of action is thought to be the binding of MLs to invertebrate glutamate-gated chloride ion channels, potentiating the influx of chloride ions, resulting in an inhibitory effect which paralyzes the associated musculature of nematodes [10, 11]. A more recent hypothesis for the mechanism of action of MLs against filarial nematodes is that MLs interfere with their ability to produce secretory products to evade the host’s immune system, resulting in host-mediated clearance of the parasite, which is likely the primary cause of nematode death [11–15]. Since oral ivermectin (Heartgard®) was introduced to the market in 1987, various formulations (oral, topical or injectable) of different drugs of the ML class have been developed for commercial use in the prevention of heartworm disease [2, 16, 17].

While MLs have remained highly effective in preventing heartworm disease in companion animals over the past 30 years, the recognition of ML resistance in the *D. immitis* population has led to renewed challenges in the prevention of this disease [18, 19]. The resistance of various isolates of *D. immitis* to MLs has been confirmed in the laboratory setting [20–24]. While these isolates are resistant to all drugs within the ML class, moxidectin has repeatedly demonstrated higher efficacy among the commercially available MLs [21, 25–27]. Moxidectin exhibits the highest lipophilicity, allowing for longer half-life, higher AUC and tissue distribution, and lower rate of clearance in comparison to the other MLs [28, 29]. Physiochemical and pharmacokinetic properties are important in modulating the rate of drug exchange between the blood stream and tissues and likely explain moxidectin’s improved performance against known ML-resistant *D. immitis* isolates compared to other MLs.

To recommend the best possible protection using currently available products, treatment protocols for dogs require careful evaluation and optimization. Veterinarians must consider the risk for individual patients (lifestyle, geographic location, owner compliance) and weigh the different options for prevention, including dosage, dosage form and treatment regimen. Knowledge of how moxidectin performs against resistant isolates using different treatment protocols may aid veterinarians in selecting the most appropriate preventive for an individual dog.

The data presented in this article provide additional information on the efficacy of oral moxidectin against JYD-34, a known ML-resistant *D. immitis* isolate, when using various dosing regimens.

## Methods

Dogs from all studies were managed similarly and with due regard for their well-being. Each study design was reviewed and approved by the sponsor's and local institutional animal care and use committees and met USDA-APHIS animal welfare requirements. All five studies were conducted in accordance with good scientific practices. Standards included masking of personnel and randomization to treatment groups.

### Animals and husbandry

In total, 52 healthy, purpose-bred Beagle dogs with no history of treatment with MLs were included in five laboratory studies. Dogs were housed in mosquito-proof, environmentally controlled facilities. All dogs underwent a full physical examination and were determined to be negative for adult *D. immitis* infection using antigen^1^ and modified Knott blood tests prior to inoculation with *D. immitis* larvae. An additional *D. immitis* antigen test was performed on Day 89 or 90 (119–120 days post-infection) to detect possible pre-existing infections present at the start of the study but not detectable at that time. All dogs were negative for heartworm antigen on Day 89 or 90 (119–120 days post-infection) confirming that they were not infected *with D. immitis* prior to the induced infections. Dogs were observed daily, from the start of acclimation to the end of the study, for general health and for adverse reactions.

### Study design

All five laboratory studies followed a randomized, negative control design. Animals were randomly allocated to study groups according to pre-treatment body weight within sex for each study. Personnel collecting safety and efficacy data were masked to treatment group. Dogs were acclimated to study conditions for at least 7 days prior to the induced *D. immitis* infection. On Day −30, each dog was inoculated with 50 *D. immitis* third-stage infective larvae (L_3_) by subcutaneous injection in the inguinal area. The studies were conducted over the course of several years as described in Table 1.

**Table 1:** Overview of study design in five studies evaluating the efficacy of treatment with moxidectin against the JYD-34 isolate of *Dirofilaria immitis*

Study no.^1^	Calendar year of execution	Group	No. of dogs per group	Treatment^2^	Dose (µg/kg)	Day(s) of treatment^3^	Days post infection of necropsy	No. of dogs with worms	Worm count range	Worm count geometric mean	% Efficacy	*P*-value^4^
1	2013	A	4	Control	N/A^5^	N/A	148	4	30–37	32.9	N/A	N/A
		B	4	Moxidectin	30	0, 30, 60	148	2	0–3	0.7	97.9	0.0030
		C	4	Moxidectin	50	0, 30, 60	148	1	0–2	0.3	99.0	0.0009
		D	4	Moxidectin	100	0	148	4	1–9	2.9	91.1	0.0133
2	2017	A	5	Control	N/A	N/A	180	5	8–29	15.2	N/A	N/A
		B	4	Moxidectin	9	0, 30, 60, 90, 120	180	1	0–1	0.2	98.8	< 0.0001
3	2017	A	5	Control	N/A	N/A	176	5	32–39	34.5	N/A	N/A
		B	4	Moxidectin	9	0, 31, 61, 90, 119	176	4	1–6	2.0	94.1	0.0025
4	2017	A	5	Control	N/A	N/A	181	5	23–51	35.9	N/A	N/A
		B	4	Moxidectin	6	0, 31, 60, 90, 119	181	4	1–14	6.0	83.3	0.0277
5	2018	A	5	Control	N/A	N/A	180	5	29–48	38.0	N/A	N/A
		B	4	Moxidectin	6	0, 31, 60, 90, 119^6^	180	4	1–6	3.7	90.2	0.0021

^1^No. = Number

^2^Control dogs were not treated

^3^All dogs were inoculated on Day −30 for each study

^4^*P*-value = Two-sided probability value from analysis of variance on log counts of moxidectin and control at α = 0.05

^5^N/A = not applicable

^6^One dog in the treated group did not have measurable levels of moxidectin following the Day 119 dose

### Heartworm isolate

The *D. immitis* isolate used for all infections was JYD-34, which originated in Illinois and has been maintained under laboratory conditions by TRS Labs, Inc., since July 2010 and validated in April 2011. JYD-34 larvae had undergone one mosquito-to-dog L_3_ passage for Study 1 and two mosquito-to-dog L_3_ passages for Studies 2–5. The donor dog used to supply L_3_ larvae in Study 1 was infected via transplantation with 10 male and 10 female adult JYD-34 worms, while the donor used in Studies 2–5 had been infected via inoculation of JYD-34 larvae. The isolate was not maintained under ML pressure. Mosquitos (*Aedes aegypti*; Liverpool strain) were infected with *D. immitis* via membrane feeding 15 days prior to inoculation. Collection and enumeration of L_3_ larvae were performed immediately prior to inoculation.

### Treatment details

Dogs ranged in age from 6.3–13.0 months at the beginning of acclimation and weighed 5.5–11.5 kg prior to the first (Day 0) treatment. All groups comprised both male and female dogs. The treatment groups are described in Table 1. Animals were weighed for dose calculations within 5 days prior to each treatment. All treatments were administered as oral solutions, with concentrations of moxidectin varying from 60 to 250 µg/ml. The treatments were non-commercial oral moxidectin solutions manufactured internally by Boehringer Ingelheim Animal Health as per Good Laboratory Practices. Immediately after oral administration of moxidectin solution, dogs were observed for swallowing to ensure the entire dose was consumed. Control dogs were not treated. All animals were observed at least hourly for 4 h following each treatment, in addition to the once daily health observations throughout the study.

### Description of necropsy and worm count

Animals were humanely euthanized using a combination of heparin and an approved euthanasia solution on their respective necropsy day (Table 1). At necropsy, the inoculation site (medial thigh) and abdominal and thoracic cavities were opened and examined for worms. The heart and lungs were removed as a unit and dissected for recovery of worms. Recovered worms were assessed for viability, sex determined and counted by masked personnel. Heartworm fragments, if any, were counted as follows: worm fragments containing a head and worm fragments containing a tail were counted separately. The greater of the two counts (number of fragments containing a head or number of fragments containing a tail) were included in the total worm count for effectiveness calculations.

### Data analysis

The total counts of live *D. immitis* were transformed to the natural logarithm of (count + 1) to calculate the geometric means for each treatment group. The efficacy of moxidectin against *D. immitis* was determined for each study by calculating the percent efficacy as 100 × [(C-T)/C], where C was the geometric mean among control animals and T was the geometric mean among moxidectin-treated animals.

For each study the treated group was compared to the control group. The MIXED procedure in SAS, version 9.4, was used for the analysis with the treatment groups listed as a fixed effect. All testing was two-sided with significance level α = 0.05.

## Results

The live, adult worm counts and geometric means from each study are listed in Table 1. In all five studies, all control dogs harbored a minimum of eight worms at necropsy. The worm recoveries in the control dogs indicated the inoculations were robust, with geometric means > 32 worms for all studies, except Study 2, which had moderate worm counts with a mean of 15.2. All moxidectin-treated groups had significantly lower (*P* ≤ 0.0277) *D. immitis* counts than control group animals for all studies (Table 1). Efficacy of moxidectin against JYD-34 ranged from 83.3% to 99.0% across the five studies presented here (Table 1).

The efficacy of both single and repeated high doses of moxidectin were explored in Study 1. In Group 1D, a single dose of 100 µg/kg was administered to the treated dogs, resulting in an efficacy of 91.1%, with 4 of 4 dogs harboring worms. In the same study, lower doses of moxidectin (30 and 50 µg/kg) were administered to dogs in Groups 1B and 1C, respectively, but dogs were treated three times at monthly intervals resulting in efficacies of 97.9% (2 of 4 dogs with worms) and 99.0% (1 of 4 dogs with worms) (Table 1).

Studies 2–5 investigated moxidectin dosed for five monthly administrations at either 9 µg/kg or 6 µg/kg. Dogs dosed at 9 µg/kg had efficacies of 98.8% or 94.1%, with 1 of 4 and 4 of 4  dogs harboring worms (Groups 2B and 3B) (Table 1). In Studies 4 and 5, animals were dosed with 6 µg/kg, resulting in efficacies of 83.3% and 90.2% (Groups 4B and 5B, respectively), with 4 of 4 dogs harboring worms in both studies (Table 1).

One dog in Study 5 did not have measurable blood levels of moxidectin after the fifth and final (Day 119) dose (Table 1, plasma data measured but not reported herein). This animal had five worms at necropsy, which was consistent with the worm counts in the other treated dogs in this study (range 1–6 worms) (Table 1). Moxidectin was measurable after all previous treatments, confirming that this dog received at least four treatments, and as its worm recovery was consistent with the other treated animals it is included in the data set.

### Health abnormalities

Treatment with oral moxidectin solution was well tolerated in all studies. In Study 3, two dogs exhibited self-limiting diarrhea within 4 h after the Day 90 treatment. The diarrhea in both dogs was considered possibly related to treatment. There were no other treatment-related abnormalities and no serious adverse events.

## Discussion

Five studies were conducted to evaluate the efficacy of varying doses and number of administrations of moxidectin against the JYD-34 heartworm isolate. This isolate was chosen as it has been shown to be resistant to MLs, and there is a large amount of  efficacy data using varying doses and dosage forms of MLs, including moxidectin, against this isolate existing in the literature [25–27, 30, 31].

The five studies reported here evaluated a high single dose of moxidectin as well as lower doses administered monthly for three or five administrations. The highest dose tested was 100 µg/kg administered once (Study 1). Despite this high dose of moxidectin, single-dose efficacy was only 91.1% with all four dogs harboring worms (range 1–9). In contrast, three monthly treatments of either 50 µg/kg or 30 µg/kg provided 99.0% and 97.9% efficacy, respectively (Study 1). The higher efficacy results following repeated administrations of a lower dose of moxidectin suggests that longer or more sustained exposure to moxidectin is more important to achieving efficacy against JYD-34 larval stages than a higher single dose.

Based on the results of Study 1, subsequent studies explored the impact of further lowering the moxidectin dose while simultaneously increasing the number of administrations (Table 1). Five monthly administrations of 9 µg/kg provided 98.8% and 94.1% efficacy (Studies 2 and 3, respectively), while 6 µg/kg administered monthly for five months provided 83.3% and 90.2% efficacy (Studies 4 and 5, respectively). These results indicate that a dose of 9 µg/kg administered for 5 months provides higher efficacy than a single 100 µg/kg dose, while 6 µg/kg seems to provide lower efficacy against JYD-34 than a single 100 µg/kg dose even when five monthly treatments are administered.

The data from these five studies suggest that oral monthly treatments with moxidectin doses as low as 9 µg/kg may be as effective as a much higher dose given only once. While biological variation could account for some differences between studies, there is a consistent trend across all studies of increasing number of doses leading to increased efficacy.

The findings reported here are consistent with reports in the literature of moxidectin efficacy against the JYD-34 isolate [26, 31]. McTier et al. [26] showed that increasing the dose of a single treatment with moxidectin can improve efficacy, as 24 µg/kg administered once provided 53.2% efficacy compared with 19.0% efficacy after a single 3 µg/kg dose. The McTier results also indicated that increasing the number of monthly doses increased efficacy, with efficacy improving from 19.0% to 44.4% after a 3 µg/kg dose was administered once or three times, respectively [26]. This same trend was seen at higher doses, as the efficacy of 24 µg/kg increased from 53.2% after a single administration to 98.8% after three monthly doses [26]. In another study, Kryda et al. [31] reported that moxidectin administered at 24 µg/kg for 4 or 6 monthly doses resulted in efficacy of 95.9% or 99.3%, respectively. These results are consistent with the data provided herein and further support the importance of both increasing dose and repeated administrations on the efficacy of moxidectin on ML-resistant heartworm isolates.

Despite the similarities seen between the current results and the McTier publications, there were also some notable differences. McTier et al. [26] reported 100% efficacy against JYD-34 with three monthly administrations of 40 µg/kg moxidectin, while 30 µg/kg and 50 µg/kg administered for three months provided 97.9% and 99% efficacy in the studies reported here (Study 1). These discrepancies may be due to normal biological variation, passage of the L_3_ larvae, limitations of small sample size or impact of infection rate in the control group. The McTier study had control takes ranging from 5–33 with a geometric mean of 20.6 worms, while our Study 1 had control takes ranging from 30–37 with a geometric mean of 32.9 worms [26].

Multiple factors may be involved to explain the better efficacy of repeated lower doses of moxidectin, such as sustained drug levels (either in the bloodstream or tissues) following multiple administrations, body fat levels, improved drug absorption and distribution in the body, or time that *D. immitis* larvae are exposed to moxidectin above a threshold level. As recent evidence hypothesizes that MLs likely do not kill *D. immitis* larvae directly but rather interfere with the larvae’s ability to escape the host immune response, it is possible that multiple doses provide more opportunities for the immune system to recognize and attack the larvae [12–15].

The data presented here are subject to several limitations, including comparison of results across different studies and small sample size increasing variability and rendering statistical analysis less meaningful. Results from studies conducted with different control groups should be interpreted with caution, as the infection rate in the control group will impact calculations of efficacy and the susceptibility of the individuals in the infective larval population may vary slightly between infections. However, because all studies reported had similar study design and used the same isolate of JYD-34, it was considered appropriate to assess the trends in efficacy across the studies.

Because these were early phase studies, four or five dogs were included per study group to minimize the number of animals used. While the group sizes did not meet the requirements outlined in VICH GL19 (six dogs per group), all control groups had a 100% infection rate with a range of recovered adult *D. immitis* worms of 8–51 and thus met the minimum recommendations for adequacy of infection as outlined by VICH GL19, i.e., harboring at least five live adult *D. immitis* for the control group dogs [32, 33]. Infection rates in the control groups were similar across Studies 1, 3, 4 and 5, with all dogs harboring 23–51 worms and geometric means ≥ 32.9. Worm counts were adequate, but somewhat lower in Study 2, with counts ranging from 8–29 in the control dogs and a geometric mean of 15.2. The infection rates in the control groups indicate that the infections were robust and hence the data from these studies are meaningful.

## Conclusions

Moxidectin administered for five consecutive months at a dose as low as 9 µg/kg provided similar efficacy against JYD-34, an ML-resistant isolate of *D. immitis*, compared to three monthly doses of 30 µg/kg, and superior efficacy to a single dose of 100 µg/kg. These data underscore the importance of repeated administrations of moxidectin in the prevention of heartworm disease in the face of potential resistance.

## Data Availability

All relevant data supporting the conclusions of this article are included within the article.

## Abbreviations

ML: Macrocyclic lactone
JYD-34: *Dirofilaria immitis* isolate JYD-34
L_3_: Third-stage larvae

## Footnotes

1 Antigen tests used varied in the studies, as follows: Study 1: PetChek® (IDEXX Laboratories, Westbrook, ME, USA), Study 2: Heska® Solo Step® CH (Heska, Loveland, CO, USA), Studies 3–5: DiroCHEK® (Zoetis, Florham Park, NJ, USA).
